# Utility of the Platelet Function Analyzer in Patients with Suspected Platelet Function Disorders: Diagnostic Accuracy Study

**DOI:** 10.1055/s-0040-1721502

**Published:** 2020-12-22

**Authors:** Jonas Kaufmann, Marcel Adler, Lorenzo Alberio, Michael Nagler

**Affiliations:** 1 Department of Hematology and Central Hematology Laboratory, Inselspital, Bern University Hospital, and University of Bern, Bern, Switzerland; 2 Division of Hematology and Central Hematology Laboratory, CHUV, Lausanne University Hospital and University of Lausanne, Lausanne, Switzerland; 3 Faculty of Biology and Medicine, University of Lausanne, Lausanne, Switzerland; 4 University Institute of Clinical Chemistry, Inselspital, Bern University Hospital, University of Bern, Bern, Switzerland

**Keywords:** platelet function analyzer, Glanzmann thrombasthenia, platelet rich plasma, Bernard-Soulier syndrome, predictive value

## Abstract

**Introduction** The platelet function analyzer (PFA) is widely used as a screening tool for bleeding disorders in various settings. The diagnostic performance regarding platelet function disorders (PFDs), which are among the most common inherited bleeding disorders, is however still elusive. We aimed to assess the diagnostic value of PFA for PFD in clinical practice.

**Methods** Comprehensive clinical and laboratory data of all consecutive patients referred to a specialized outpatient between January 2012 and March 2017 with a suspected bleeding disorder were prospectively recorded. The diagnostic work-up was performed according to a prespecified protocol following current guidelines and platelet function was tested using light transmission aggregometry as well as flow cytometry.

**Results** Five hundred and fifty-five patients were included (median age 43.7 years; interquartile range [IQR] 29.3, 61.7; 66.9% female). Possible PFD was diagnosed in 64 patients (11.5%) and confirmed PFD in 54 patients (9.7%). In patients with confirmed PFD, median closure times were 107 seconds (ADP or adenosine diphosphate; IQR 89, 130) and 169 seconds (EPI; IQR 121, 211). In patients without bleeding disorders, PFA closure times were 96 seconds (ADP; IQR 83, 109) and 137 seconds (EPI; IQR 116, 158). The sensitivity was 19.5% in case of PFA ADP (95%CI 12.6, 30.0; specificity 86.4%; 95% CI 82.4, 89.8), and 44.3% in case of PFA EPI (95% CI 34.9, 53.9; specificity 75.6%; 95% CI 70.8, 79.9).

**Conclusion** The diagnostic performance of PFA for PFD was moderate to poor. Our results do not support the utilization of PFA for screening of PFD in clinical practice.

## Introduction

Hereditary platelet function disorders (PFDs) are among the most common causes of a bleeding disorder that manifest in the context of trauma and/or medical interventions. 1 2 However, diagnosing PFD is difficult and many patients probably remain unidentified, exposing them to a risk for bleeding in the periinterventional setting. 3 Severe disorders such as Glanzmann thrombasthenia (GT), Bernard-Soulier syndrome (BSS), or syndromic disorders manifest with a moderate to severe bleeding phenotype and are rather straightforward to diagnose. 4 In contrast, the majority of PFDs are much more difficult to recognize and diagnose. Bleeding symptoms are often mild and patients can be barely distinguished from healthy individuals. Appropriate laboratory tests are available only in specialized laboratories because PFD tests are technically challenging, time-consuming, and difficult to interpret. 3 5 Because of complex preanalytic requirements, blood drawing has to be done on site at the laboratory rather than having the sample shipped from farther afield. 6 7 A screening test would be of great value in the work-up of patients with suspected PFD.

Due to its simplicity of use and a high sensitivity with regard to several factors affecting primary hemostasis, the PFA has been implemented widely in the work-up of patients with a suspected bleeding disorder. 8 9 Some authors have considered the so-called “in vitro bleeding time” to be at least as sensitive as the light transmission aggregometry approach and even more sensitive than the skin bleeding time (IVY method). 8 9 10 In addition, PFA has been suggested as a preoperative screening tool 11 12 and several studies have confirmed a very high sensitivity with regard to von Willebrand disease. 13 In addition, PFA is an excellent means to monitor the treatment with desmopressin in patients with von Willebrand disease. 14 Moreover, PFA is very sensitive for the presence of aspirin. 15 Data are much more heterogenous with regard to PFD. 8 PFA appears to detect all patients with a severe PFD such as GT or BSS. 8 10 13 Only a few studies have investigated the diagnostic accuracy with regard to mild PFD and the results vary considerably. 8 A large prospective study conducted in a representative, unselected cohort of patients with a broad range of PFD is however still missing. Thus, whether or not PFA can be used as a screening tool for PFD remains unclear.

With the present study, we aimed to assess the diagnostic value of the PFA for a variety of PFD in a large representative cohort of patients.

## Methods

### Study Design, Setting, and Population

All consecutive patients aged 18 years or older, referred between January 2012 and March 2017 to our specialized outpatient unit of Inselspital University Hospital with a suspected bleeding disorder were included and data were prospectively recorded. The study population is regarded as representative for Switzerland for the following reasons: the Inselspital is a tertiary university hospital covering a region with 1.5 million inhabitants; the greater Bern area comprises rural as well as urban communities; and the canton of Bern has both French-speaking and German-speaking regions. The outpatient unit of Inselspital is a reference center for coagulation disorders and contains the only laboratory within the described area providing a comprehensive set of laboratory assays. The vast majority of patients with a suspected bleeding disorder will therefore be referred to our institution. Reasons for referral included (1) a bleeding tendency, (c) a family history of bleeding disorders, or (d) abnormal laboratory test results. The methodology has been previously described. 16

The clinical data were prospectively collected using an established in-house questionnaire as well as the ISTH-BAT sheet and stored securely in the electronic hospital database. Laboratory results were saved accordingly. The patient records were coded using an in-house identification system and data were retrieved by two investigators in parallel (M.A./J.K., M.N.). All patients signed informed consent and the Ethics Committee approved the study protocol (No. 02289).

### Work-up of Patients

Following previous recommendations, a standardized protocol was created and followed in the diagnostic work-up of patients. 5 17 18 19 20 First, patients completed a survey focused on bleeding history and antithrombotic drugs, which was provided by mail in advance of the consultation. 21 Second, comprehensive bleeding history was taken by trained resident physicians using a standardized 13-item, in-house questionnaire assessing the presence and severity of bleeding at specific organs, including the skin, nose, oral cavity, gastrointestinal, urogenital, joints and muscles, bleeding in association with minor injuries, dental procedures, surgery, transfusion requirements, bleeding after ingestion of drugs known to affect hemostasis and family history, 21 and the ISTH-BAT form was completed. 17 22 A physical examination was performed, focusing on hematomas or petechia, signs of amyloidosis, telangiectasia, as well as hyperflexibility of joints. Instructions were given to stop antithrombotic treatment, selective serotonin reuptake inhibitors, and nonsteroidal anti-inflammatory drugs 10 days prior to investigation. Patients were additionally instructed to refrain from smoking and drinking caffeine on the day of consultation.

### Handling of Samples

Blood drawing and preparation of blood samples was done following a standardized protocol in order to avoid preanalytical errors. 23 Blood samples were collected by standard venepuncture with a 21-gauge needle. EDTA (ethylenediaminetetraacetic acid) tubes (Monovette; Sarstedt, Nümbrecht, Germany) were used for determination of full blood count, citrated tubes (0.106 mol/L trisodium citrate 9:1, v/v) for the determination of coagulation tests and flow cytometry, as well as buffered citrate (0.13 mol/L trisodium citrate, pH 5.5 corresponding to 3.2% trisodium citrate) for platelet aggregometry as well as PFA. Samples were transported manually to the laboratory. Samples were snap-frozen at −80°C for some assays conducted in batches. Platelet rich plasma (PRP) was prepared by centrifugation at room temperature for 15 minutes at 150 g. Platelet poor plasma was prepared by centrifugation at 1,500 g for 15 minutes.

### Laboratory Work-up

A stepwise approach was used in the laboratory work-up. The following initial tests were carried out simultaneously: full blood count and mean platelet volume, a blood smear analyzing platelet morphology, prothrombin time, activated partial thromboplastin time, thrombin time (TT), fibrinogen concentration (Clauss' method), α2-antiplasmin, a range of coagulation factors (FII, FV, FVII, FX, FXIII), von Willebrand factor (VWF) antigen (VWF:Ag), VWF activity (VWF:GPIbM), and platelet function analyzer (PFA-200). The determination of light transmission aggregometry (LTA), flow cytometry, and PFA-200 is described in below, all other tests are characterized in the Supplementary Material. The following tests were conducted in few selected cases: chromogenic factor VIII, VWF multimer analysis, VWF-FVIII binding capacity, lumi-aggregometry, and molecular diagnostics.

### Determination of Platelet Function Analyzer

PFA was performed as previously described using a microprocessor-controlled instrument (PFA-200 and INNOVANCE PFA-200 System, Siemens Healthcare Diagnostics, Germany) and a disposable test cartridge. 21 For all patients, two cartridges were used (Dade PFA collagen/epinephrine [designated here as EPI] and Dade PFA collagen/ADP [designated here as ADP], Siemens Healthcare Diagnostics, Germany). The closure times (CTs), defined as the time to full occlusion of the aperture, were measured for both cartridges. The established in-house reference ranges were 65 to 130 seconds for ADP and 80 to 170 seconds for EPI.

### Determination of Light Transmission Aggregometry and Platelet Flow Cytometry

We conducted LTA in-line with current recommendations 7 24 and as previously described. ^21^ The aggregometer APACT 4004V (LABiTec GmbH, Ahrensburg, Germany) was used. Baseline agonists for platelet aggregation were ADP (Sigma-Aldrich, St. Louis, Missouri, United States; 4, 6, and 10 μmol/L for male patients and 3, 4, and 6 μmol/L for female patients), collagen (HORM; Nycomed, Linz, Austria) at 1.5, 3, and 4 μg/mL, arachidonic acid at 2 mmol/L (Bio Data/Medonic Servotec AG, Interlaken, Switzerland), and ristocetin at 1.5 and 0.5 mg/mL (Socochim SA, Lausanne, Switzerland). Platelet count was adjusted to 250 × 10 ^9^ /L. In order to exclude spontaneous aggregation, 200 μL of PRP prewarmed at 37°C for 1 minute, was added to the aggregometer cuvette and run for an additional minute. After adding 20 μL of the agonist to the sample, the response was recorded. In case of a response to one agonist outside the limits of the normal range, the test was repeated. LTA was performed between 1 and 2.5 hours after collection of venous blood samples from the patient. Previously established in-house reference values were used for interpretation of the aggregation curves. 25 As an internal control, a sample from a healthy volunteer was analyzed. LTA was not performed when the platelet count of the patient was <100 G/L.

Platelet flow cytometry was conducted as previously described. 16 21 The following antihuman antibodies were used for analysis of surface glycoproteins (GPs): Ibα (monoclonal antihuman CD42b-PE; Dako, Baar, Switzerland), GPIIb/IIIa (anti-hCD41-FITC and anti-hCD61-FITC, Becton Dickinson, Allschwil, Switzerland), baseline P-selectin expression (anti-CD62P-PE, Becton-Dickinson), and PAC-1 binding (PAC1-FITC, Becton Dickinson). After preparation of the sample in a 100 μL volume containing platelets at a concentration of 5 × 10 ^6^ /mL and anti-CD62P-PE and PAC1-FITC, analysis was performed with a FACSCanto (Becton Dickinson, Heidelberg, Germany) flow cytometer. By using ADP (0.5, 5.0, and 50 μmol/L), convulxin (5, 50, and 500 ng/mL), and thrombin (0.05, 0.5, and 5 μmol/L), the dose response of platelet reactivity was investigated. The surface expression of negatively charged phospholipids was investigated using Annexin V-FITC (Roche, Rotkreuz, Switzerland) after incubation with either Ionophore A 23187 or the combination of convulxin (500 ng/mL) and thrombin (5 nmol/L). By loading platelets with mepacrine (at 0.17 μmol/L as well as 1.7 μmol/L) and analyzing with thrombin, the content and secretion of dense granules were evaluated. The in-house reference values had been previously established. 21 A sample from a healthy volunteer was analyzed in parallel with each run. To confirm the results, flow cytometric analysis was repeated once with different control platelets.

### Definition of Diagnoses

Bleeding disorders were diagnosed in line with recent guidelines and recommendations; the bleeding history was considered in addition to laboratory tests. Abnormal laboratory results were repeated at least once in order to exclude spurious results.

PFD were categorized into “possible” or “confirmed” PFD regarding whether or not all platelet function studies were available. “Confirmed PFDs” were defined as an abnormal light transmission aggregometry and/or an abnormal platelet flow cytometry on two occasions in the absence of other disorders. 5 6 Patients were categorized with a “possible PFD” if only one measurement was available or there were inconclusive results, or concomitant disorders were present. PFDs were further categorized into eight subgroups: (1) Glanzmann's thrombasthenia, defined as a defect in GPIIb/IIIa associated with a severely diminished aggregation of all agonists except ristocetin, reduced expression of GPIIb/IIIa, and/or markedly reduced activation of PAC1-binding 1 3 4 26 ; (2) Gi-like defects, defined as an accentuated deficiency in aggregation to the Gi-coupled receptor antagonists ADP and adrenaline, associated with corresponding flow cytometry results, 1 3 26 (3) thromboxane A _2_ pathway defects, defined as an absent aggregation in response to arachidonic acid, and possibly associated with an impaired response to other agonists, 1 3 4 24 26 (4) dense granule secretion defects, defined as a defect in storage and/or secretion of mepacrine, 1 3 4 21 26 (5) collagen receptor defects, defined as an isolated reduction in aggregation and secretion after stimulation with collagen and convulxin, 1 21 26 (6) α-granule disorders, defined as a reduced expression and/or secretion of P-selectin, associated with varying impaired aggregation after stimulation with collagen and epinephrine, 1 21 (7) decreased generation of procoagulant COAT platelets, defined as an impaired binding of Annexin-V after incubation with convulxin and thrombin, 21 (8) complex disorders, defined as defects in a number of agonists (LTA) and/or several flow cytometry results that cannot be attributed to any of the disorders mentioned above. LTA and flow cytometry were interpreted according to previous recommendations and established in-house reference ranges. 21 Three experienced individuals performed the analyses and discrepancies were resolved by discussion. 3 5 6 27 28 29 Additionally, lumi-aggregometry was considered if available (for a few patients only).

VWD type 1 was diagnosed with VWF:GPIbM levels of 0.05 to 0.4 U/mL and VWF:Ag of 0.05 to 0.4 U/mL, an VWF:GPIbM/ VWF:Ag ratio of >0.7, a normal multimer pattern and a relevant bleeding history (repeated measurements). 30 31 32 33 34 Aiming to simplify treatment decisions in clinical practice, we defined a threshold of 0.4 U/mL rather than a 0.3 U/mL. 35 VWD type 2 was diagnosed following ISTH criteria. 32 Low VWF was diagnosed in patients with VWF:GPIbM or VWF:Ag below 0.5 U/mL, not meeting the criteria mentioned above, and associated with blood group O. 19 Hemophilia and other single factor deficiencies were diagnosed in accordance with current definitions. 36

We categorized a patient with an abnormal ISTH-BAT (male ≥4 points; female ≥6 points) as a “bleeder of undefined cause” if all other tests mentioned were normal and no bleeding disorder was identified. 37 38 39 Patients with a systemic disorder associated with bleeding symptoms (e.g., hereditary telangiectasia and thrombocytopenia) but without a hemostatic were categorized as “systemic disorders.”

### Statistical Analysis

The study population was characterized using descriptive statistics (numbers/percent or median/interquartile range [IQR] as appropriate). The ability of the PFA to discriminate between healthy individuals and patients with PFD (suspected or confirmed) was assessed with a receiver-operating characteristic (ROC) curve analysis. Sensitivity and specificity were calculated using the in-house reference values and the reference values of the manufacturer (as a sensitivity analysis). Stata 14.2 statistics software package was used (StataCorp. 2014. Stata Statistical Software: Release 14 College Station, Texas: StataCorp LP) and figures were created using Prism 8 (GraphPad Software, Inc., La Jolla, California, United States).

## Results

### Patient Characteristics

**Fig. 1:**
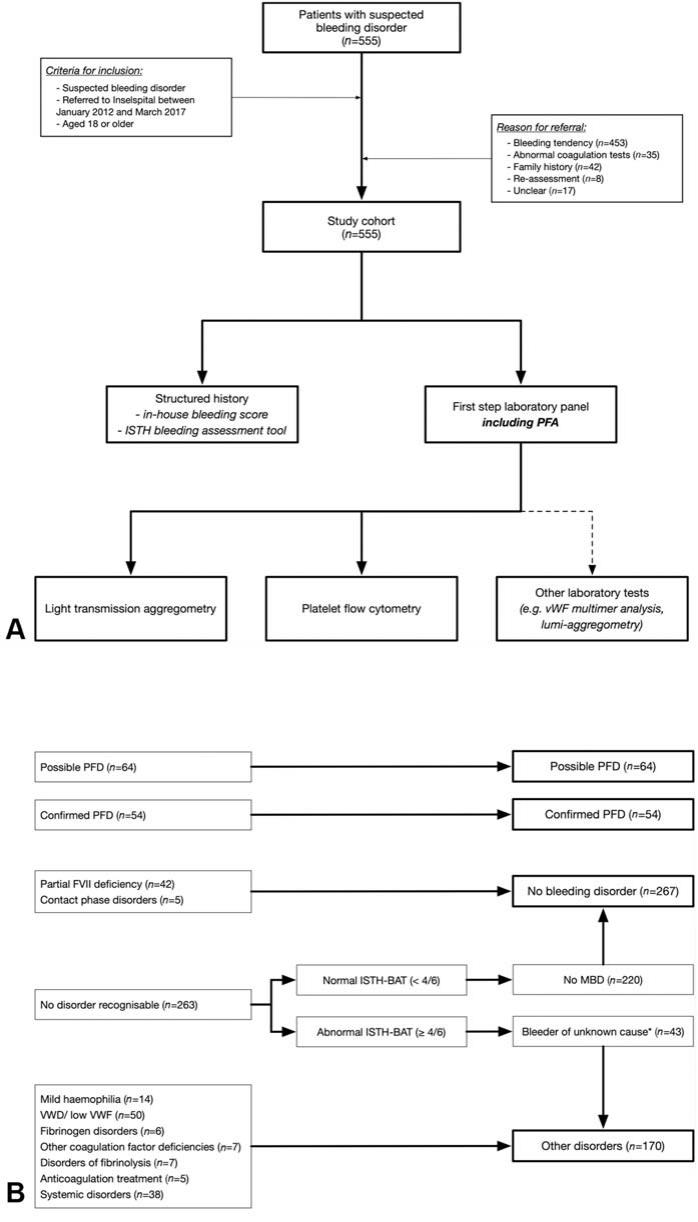
( **A** ) Patient flow. In a prospective cohort study, we collected clinical characteristics and laboratory data from all consecutive patients referred between January 2012 and March 2017 to an outpatient unit of a university hospital with a suspected bleeding disorder. The diagnostic work-up was performed following current guidelines and platelet function was tested using light transmission aggregometry as well as flow cytometry. ( **B** ) Classification of patients according to diagnosis. PFD, platelet function disorder; MBD, mild bleeding disorder; VWD, von Willebrand disease; VWF, von Willebrand factor; FVII, factor VII.

Five hundred and fifty-five patients were referred with a suspected bleeding disorder between January 2012 and March 2017 and included in the study cohort ( Fig. 1A ). The median age was 43.7 years (IQR 29.3, 61.7) and 371 patients were female (66.9%). Referrals were made by primary care physicians in 200 cases (36.0%), gynecologists in 121 (21.8%), and other medical specialists in 207 cases (37.3%). The most common reason for referral was a bleeding tendency in 453 cases (81.6%), followed by abnormal coagulation tests in 35 cases (6.3%), a family history in 42 cases (7.6%), and verification of a known hemostatic disorder (re-evaluation) in eight cases (1.4%). Antiplatelet drugs were used by 54 patients (9.7%, stopped 10 days before assessment), 34 patients were being treated with anticoagulant drugs (6.1%, predominantly vitamin K-antagonists) and 35 patients (6.3%) received selective serotonin reuptake inhibitors. The ISTH-BAT was abnormal (male ≥4 points; female ≥6 points) in 153 patients referred for a bleeding tendency (35%), and in 156 patients referred for any reason (28.1%). Detailed patient characteristics of the study population are shown in Table 1 .

**Table 1:** Characteristics of patients referred with a suspected bleeding disorder ( *n* = 555; 2012–2017; described in Adler et al 16 )

Characteristics	No bleeding disorder	Possible platelet function disorder a	Confirmed platelet function disorder a	Other bleeding disorders	Missing data
	Numbers (%) or median (IQR) as appropriate				
Patients	267 (48.0)	64 (11.5)	54 (9.7)	170 (30.6)	0
Age (years)	40.2 (27.3, 60.4)	49.3 (34.9, 63.9)	49.8 (33.5, 64.0)	44.3 (30.7, 61.7)	0
Sex					
Female	186 (69.7)	52 (74.3)	32 (66.7)	101 (59.4)	0
Male	81 (30.3)	18 (25.7)	16 (33.3)	69 (40.6)	0
Reason for referral					17
Bleeding tendency	191 (75.5)	63 (98.4)	51 (94.4)	148 (88.6)	
Abnormal coagulation tests	25 (9.9)	1 (1.6)	0 (0)	9 (5.4)	
Family history	33 (13.0)	0 (0)	3 (5.6)	6 (3.6)	
Re-evaluation	4 (1.6)	0 (0)	0 (0)	4 (2.4)	
Referring physician					27
Primary care physician	90 (35.9)	22 (32.8)	20 (42.6)	68 (41.7)	
Gynecologist	51 (20.3)	18 (26.9)	18 (38.3)	34 (20.9)	
Other specialist	110 (43.8)	27 (40.3)	9 (19.2)	61 (37.4)	
ISTH-BAT score	2 (1,3)	4 (2, 7)	7 (5, 9)	4 (2, 7)	15
Antiaggregant treatment	23 (9.5)	11 (17.2)	3 (6.8)	17 (10.8)	47
Anticoagulant treatment	13 (4.9)	3 (4.7)	2 (3.7)	16 (9.5)	3
SSRI treatment	16 (6.7)	8 (14.3)	4 (8.3)	7 (4.1)	57
VWF antigen	110 (82, 136)	101 (79, 136)	98 (79, 133)	93 (57, 134)	72
VWF activity	107 (81, 131)	99 (72, 126)	100 (75, 137)	84 (55, 126)	73
Platelet count	235 (200, 267)	256 (223, 307)	235 (186, 270)	227 (184, 273)	4

Abbreviations: IQR, interquartile range; SSRI, selective serotonin uptake inhibitors; VWF, von Willebrand factor; ISTH-BAT, bleeding assessment tool of the International Society on Thrombosis and Hemostasis.

a Diagnosis of a platelet function disorder was made using light transmission aggregometry and platelet flow cytometry. “Confirmed platelet function disorder” was defined as abnormal results in repeated latent transition analysis/flow cytometry measurements in the absence of other disorders, “possible platelet function disorder” as an abnormal result in one measurement available, inconclusive results, or presence of concomitant disorder.

### Type of Bleeding Disorders

Two hundred and eighty-eight out of 555 patients were diagnosed with a bleeding disorder (51.9%; Table 1 ); the type of disorder is reported in Fig. 1B . A possible PFD was identified in 64 patients (11.5%) and a confirmed PFD in 54 cases (9.7%). Von Willebrand disease (or low von Willebrand factor) was diagnosed in 50 cases (9.0%), mild hemophilia in 14 cases (2.5%), deficiency of other coagulation factors in seven cases (1.3%; including six patients with factor XI deficiency and one patient with factor X deficiency), fibrinogen disorders in six cases (1.1%), disorders of fibrinolysis in seven cases (1.3%; including one patient with α2-antiplasmin deficiency, three patients with PAI1 deficiency and three patients with abnormal clot lysis time), anticoagulant treatment in five cases (0.9%), and a systemic disorder in 38 cases (6.9%).

### Subgroups of PFD

PFDs were categorized into subgroups according to their laboratory test results ( Table 2 ). Gi-like defects were diagnosed in 38 patients (32.2%), complex disorders in 33 cases (28.0%), TxA2 pathway defects in 14 (11.9%), diminished procoagulant COAT platelets in 10 (8.5%), collagen receptor defects in eigth (6.8%), α-granule disorders in six (5.1%), dense granule disorders in five (4.2%), and Glanzmann thrombasthenia in four patients (3.4%). The distribution according to the diagnostic group (possible vs. confirmed) is shown in Table 2 .

**Table 2:** Platelet function disorder subgroups (described in Adler et al 16 )

Disorder	Possible PFD ( *n* = 64)	Confirmed PFD ( *n* = 54)
	Numbers (%)	
Glanzmann's thrombasthenia a	0	4 (7.4)
Gi-like defects b	20 (31.3)	18 (33.3)
TxA2 pathway defects c	13 (20.3)	1 (1.9)
Collagen receptor defects d	6 (9.4)	2 (3.7)
Dense granule disorders e	1 (1.6)	4 (7.4)
α-granule disorders f	1 (1.6)	5 (9.3)
Diminished procoagulant COAT platelets g	3 (4.7)	7 (13.0)
Complex disorders h	20 (31.3)	13 (24.1)

Abbreviations: ADP, adenosine diphosphate; PFD, platelet function disorders; TxA2, thromboxane A2. PFD subgroups were defined as follows.

a Defect in GPIIb/IIIa associated with a severely diminished aggregation of all agonists except ristocetin, reduced expression of GPIIb/IIIa, and/or markedly reduced activation of PAC1-binding. 1 3 4 26

b Accentuated deficiency in aggregation to the Gi-coupled receptor antagonists ADP and adrenaline, associated with corresponding flow cytometry results. 1 3 26

c Absent aggregation in response to arachidonic acid, and possibly associated with an impaired response to other agonists. 1 3 4 24 26

d Isolated reduction in aggregation and secretion after stimulation with collagen and convulxin. 1 21 26

e Defect in storage and/or secretion of mepacrine. 1 3 4 21 26

f Reduced expression and/or secretion of P-selectin, associated with varying impaired aggregation after stimulation with collagen and epinephrine. 1 21

g Impaired binding of Annexin-V after incubation with convulxin and thrombin. 21

h Defects in a number of agonists (LTA) and/or several flow cytometry results that cannot be attributed to any of the disorders mentioned above.

### PFA Closure Times According to Diagnosis

PFA (ADP, EPI) was conducted in 473 out of 555 patients ( Table 3 ). Data were missing for one patient with a possible PFD and in four patients with a confirmed PFD—because PFA was performed in the referring laboratory.

**Table 3:** Closure times of PFA according to disease category in a cohort of patients referred with a suspected bleeding disorder ( *n* = 555)

Disorder	PFA (ADP)	PFA (EPI)	Number of patients	Missing data
	Median closure time in seconds (IQR)			
No bleeding disorder	96 (83, 109)	137 (116, 158)	267	48
Possible platelet function disorder a	101 (89, 124)	157 (128, 219)	64	1
Confirmed platelet function disorder a	107 (89, 130)	169 (121, 211)	54	4
VWD/low VWF b	149 (122, 199)	206 (160, 300)	50	1
Other disorder c	107 (88, 140)	158 (131, 216)	120	28

Abbreviations: EPI, epinephrine; IQR, interquartile range; LTA, light transmission aggregometry; VWD, von Willebrand disease; VWF, von Willebrand factor.

a Diagnosis of platelet function disorder was determined using light transmission aggregometry and platelet flow cytometry. “Confirmed platelet function disorder” was defined as abnormal results in repeated LTA/flow cytometry measurements in the absence of other disorders, “possible platelet function disorder” as abnormal result in one measurement available, inconclusive results, or presence of concomitant disorders.

b Low von Willebrand values associated with blood group 0; von Willebrand disease type 1 or type 2.

c Systemic disorder associated with bleeding symptoms such as hereditary hemorrhagic telangiectasia and thrombocytopenia.

**Fig. 2:**
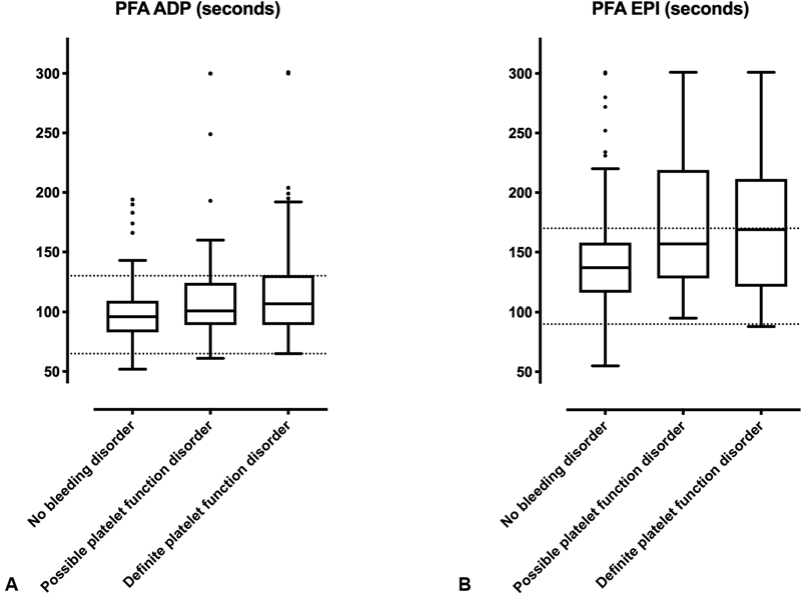
Closure times of PFA according to the presence of a platelet function disorder. PFA was conducted in a cohort of 555 consecutive patients referred with a suspected bleeding disorder. Diagnosis of platelet function disorder was achieved using light transmission aggregometry and platelet flow cytometry. We defined “confirmed platelet function disorder” as abnormal results in repeated LTA/flow cytometry measurements in the absence of other disorders and “possible platelet function disorder” with an abnormal result in one measurement available, inconclusive results or presence of concomitant disorders. LTA, light transmission aggregometry. ( **A** ) PFA ADP measurements, ( **B** ) PFA epinephrine measurements.

Median PFA CTs were 101 seconds (ADP; IQR 89, 124) and 157 seconds (EPI; IQR 128, 219) in patients with a possible PFD. In patients with a confirmed PFD, median CTs were 107 seconds (ADP; IQR 89, 130) and 169 seconds, respectively (EPI; IQR 121, 211). In patients without a bleeding disorder, PFA CTs were 96 seconds (ADP; IQR 83, 109) and 137 seconds (EPI; IQR 116, 158). Detailed results are reported in Table 3 and Fig. 2 .

### Predictive Value of PFA

**Fig. 3:**
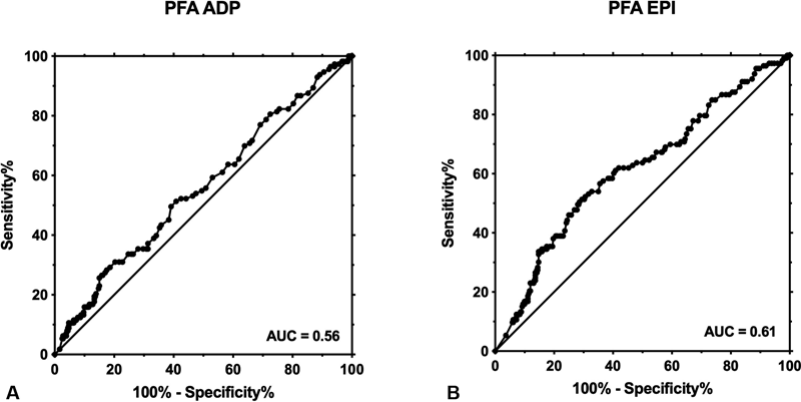
The receiver operating characteristics (ROC) curve of PFA closure times for the presence of a platelet function disorder. ( **A** ) PFA ADP: area under the ROC curve (AUC): 0.56 (95% CI 0.50, 0.62). At a threshold of 130 seconds, sensitivity was 19.5% (95% CI 12.6, 30.0) and specificity 86.4% (95% CI 82.4, 89.8). ( **B** ) PFA EPI: AUC 0.61 (0.55, 0.67). At a threshold of 170 seconds, sensitivity was 44.3% (34.9, 53.9) and specificity was 75.6% (70.8, 79.9). EPI, epinephrine; ADP, adenosine diphosphate; PFD, platelet function disorders.

The predictive value of the PFA for the presence of (suspected and confirmed) PFD is illustrated in Fig. 3 . The area under the ROC curve was 0.56 (95% CI 0.50, 0.62) in case of ADP and 0.61 in case of EPI (95% CI 0.55, 0.67). Sensitivity and specificity were calculated according to the in-house reference ranges (PFA ADP 65–130 seconds; PFA EPI 80–170 seconds). In case of PFA ADP, the sensitivity was calculated to be 19.5% (95% CI 12.6, 30.0) and specificity was 86.4% (95% CI 82.4, 89.8). The positive predictive value was 18.1% (12.4, 25.0), the negative predictive value 77.5% (73.1, 81.5), the positive likelihood ratio 0.82, and the negative likelihood ratio 1.1. In case of PFA EPI, sensitivity was 44.3% (95% CI 34.9, 53.9) and specificity was 75.6% (95% CI 70.8, 79.9), respectively. The positive predictive value was 25.5% (19.9, 31.7), the negative predictive value was 81.5% (77.0, 85.6), the positive likelihood ratio was 1.3, and the negative likelihood ratio 0.84.

We conducted a sensitivity analysis using the reference ranges of the manufacturer (PFA ADP 62–100 seconds; PFA EPI 82–150 seconds). In case of ADP, the sensitivity was 53.1% (43.5, 62.6) and the specificity was 53.6% (48.3, 58.9). With regard to EPI, the sensitivity was 61.1% (51.4, 70.1) and the specificity was 59.2% (53.9, 64.3).

## Discussion

We studied a large representative cohort of consecutive patients with a suspected bleeding disorder. We identified 64 patients with a possible PFD (11.5%) and 54 patients with a confirmed PFD (9.7%). PFA CTs were only slightly higher in patients with possible and confirmed PFD and the diagnostic performance was moderate to poor.

Previous investigations in different settings and using different study designs revealed inconsistent results. Cattaneo and colleagues measured PFA CTs in seven patients with δ-storage pool deficiency, ten patients with primary platelet secretion defect (PSD), and 40 controls. 40 Sensitivity was 41 or 47%, respectively. Harrison et al assessed the potential of the PFA in a cohort of selected patients. 41 Overall sensitivity was found to be 81% for ADP and 86% for EPI. Buyukasik et al studied PFA in a cohort of patients with PSDs and controls. 42 Sensitivity was 81.6% in this study. Quiroga et al compared the results of the PFA in patients with mucocutaneous bleeding to healthy controls. 43 The sensitivity was low for patients with isolated PSD or patients with an unknown bleeding disorder (24 and 15%, respectively). A low sensitivity was also observed in a study focusing mainly on preoperative patients. 44

There are a number of strengths to our study. We evaluated the PFA in an unselected target population comprising all patients referred with a suspected bleeding disorder. Any selection bias is prevented through the consecutive inclusion of adult Caucasian patients. The number of patients is considered high and the evaluation was done using a prespecified protocol including LTA as well as platelet flow cytometry. Several potential limitations do appear, however. First, a number of patients were reluctant to attend the outpatient unit several times, thus preventing a complete diagnostic work-up. To accommodate this, we analyzed these patients separately (possible PFD) and similar associations were observed. Second, PFA was not conduced in a number of patients for a variety of reasons, but we believe this has not introduced any bias as PFA measurements were nearly complete in patients with suspected or confirmed PFD. In addition, tests beyond LTA and flow cytometry were rarely available in our setting (e.g., molecular diagnostics, ATP release, fluorescence microscopy). Again, we do not anticipate that this introduced any bias. Third, the study population was defined as consecutive patients referred to a tertiary care center, and one might argue that this does not apply to secondary care settings where the test is usually applied. We agree with this argument, but we are nevertheless convinced that the present study represents the best level of evidence available. As long as no well-designed studies in primary care are available, we do not see good arguments for using PFA as a screening tool for PFD in clinical practice. Fourth, diagnosing PFDs is complex, and no single test covers all aspects of platelet function. 45 Besides, considerable uncertainty is introduced with every assay because of reproducibility issues and false-positive results. However, we believe that the combination of LTA and flow cytometry is a good compromise because it covers many aspects of platelet function while limiting the number of tests conducted. And, both assays are particularly recommended by current guidelines. 5

Even though PFA is a sensitive assay to detect VWD, it appears that it is not useful to detect PFD. As long as PFD are among the most frequent bleeding disorders, few arguments remain using PFD as a screening tool for bleeding disorders in clinical practice. But the question is, what should we do? We believe that taking the bleeding history using the ISTH BAT is the most useful test to screen for inherited bleeding disorders. The ISTH BAT is validated in all frequent disorders such as VWD and PFD. 16 22 29 However, some might argue that it is elaborate and complicated for the inexperienced observer. Thus, future research shall develop short and concise versions of the ISTH BAT.

In conclusion, in this large study of consecutive patients with a suspected bleeding disorder, PFA CTs were only slightly higher in patients with PFD compared to patients without a bleeding disorder and the diagnostic performance was very limited. As long as PFD remains among the most common hereditary bleeding disorders, our data do not support the implementation of PFA for the screening of bleeding disorders in clinical practice.
